# Primary resistance to nivolumab plus ipilimumab therapy in patients with metastatic renal cell carcinoma

**DOI:** 10.1002/cam4.6306

**Published:** 2023-07-05

**Authors:** Kazuyuki Numakura, Yuya Sekine, Shingo Hatakeyama, Yumina Muto, Ryuta Sobu, Mizuki Kobayashi, Hajime Sasagawa, Soki Kashima, Ryohei Yamamto, Taketoshi Nara, Hideo Akashi, Ryuji Tabata, Satoshi Sato, Mitsuru Saito, Shintaro Narita, Chikara Ohyama, Tomonori Habuchi

**Affiliations:** ^1^ Department of Urology Akita University Graduate School of Medicine Akita Japan; ^2^ Department of Urology Hirosaki University Graduate School of Medicine Hirosaki Japan; ^3^ Department of Anatomy Akita University Graduate School of Medicine Akita Japan; ^4^ Department of Urology Ageo Central General Hospital Ageo Japan

**Keywords:** ipilimumab, lymph node metastasis, neoplasm metastasis, nivolumab, renal cell carcinoma, survival rate

## Abstract

**Background:**

Nivolumab plus ipilimumab (NIVO+IPI) is the first‐line treatment for patients with metastatic renal cell carcinoma (mRCC). Approximately 40% of patients achieve a durable response; however, 20% develop primary resistant disease (PRD) to NIVO+IPI, about which little is known in patients with mRCC. Therefore, this investigation aimed to evaluate the clinical implication of PRD in patients with mRCC to select better candidates in whom NIVO+IPI can be initiated as first‐line therapy.

**Methods:**

This multi‐institutional retrospective cohort study used data collected between August 2015 and January 2023. In total, 120 patients with mRCC treated with NIVO+IPI were eligible. Associations between immune‐related adverse events and progression‐free survival, overall survival (OS), and objective response rate were analyzed. The relationship between other clinical factors and outcomes was also evaluated.

**Results:**

The median observation period was 16 months (interquartile range, 5–27). The median age at NIVO+IPI initiation was 68 years in the male‐dominant population (*n* = 86, 71.7%), and most patients had clear cell histology (*n* = 104, 86.7%). PRD was recorded in 26 (23.4%) of 111 investigated patients during NIVO+IPI therapy. Patients who experienced PRD showed worse OS (hazard ratio: 4.525, 95% confidence interval [CI]: 2.315–8.850, *p* < 0.001). Multivariable analysis showed that lymph node metastasis (LNM) (odds ratio: 4.274, 95% CI: 1.075–16.949, *p* = 0.039) was an independent risk factor for PRD.

**Conclusions:**

PRD was strongly correlated with worse survival rates. LNM was independently associated with PRD in patients with mRCC receiving NIVO+IPI as first‐line therapy and might indicate that a candidate will not benefit from NIVO+IPI.

## Graphical abstract

This study evaluated the clinical significance of primary resistant disease (PRD) in patients with metastatic renal cell carcinoma (mRCC) treated with nivolumab plus ipilimumab (NIVO+IPI) as first‐line therapy. Of the 120 patients included, 23.4% experienced PRD, which was associated with worse overall survival (hazard ratio: 4.525). Lymph node metastasis (LNM) was an independent risk factor for PRD. This study suggests that LNM could be used to identify patients who may not benefit from NIVO+IPI as first‐line therapy.

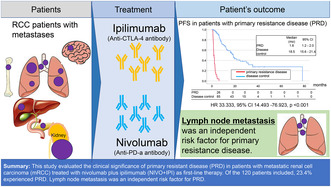

## 1 INTRODUCTION

The treatment strategy has dramatically changed since immune‐oncological (IO) drugs were approved as a treatment option for advanced renal cell carcinoma (RCC).^1^ Furthermore, a combination regimen of anti‐programmed death‐1 antibody (PD‐1) with cytotoxic T‐lymphocyte antigen‐4 (CTLA‐4) antibody or vascular endothelial growth factor receptor (VEGFR) tyrosine kinase inhibitor (TKI) has been a standard treatment for patients with metastatic RCC (mRCC) as first‐line therapy.^2^ Nivolumab (an anti‐PD‐1 antibody) plus ipilimumab (an anti‐CTLA‐4 antibody) (NIVO+IPI) is a standard treatment option for patients with mRCC at intermediate and poor risk as per the International Metastatic Renal‐Cell Carcinoma Database Consortium (IMDC).^3^ The 50% overall survival (OS) was >4 years, and 40% of the patients achieved durable responses in CheckMate 214.^4^ This survival advantage is superior to VEGFR‐TKI monotherapy. In contrast, approximately 20% of patients suffered from a primary resistant condition to NIVO+IPI, which led to a poor prognosis.^5^ Clinicians are required to identify patients for whom NIVO+IPI might not be beneficial, but most clinicians tend to prolong the NIVO+IP treatment for up to 3 months due to concern for possible pseudoprogression.^6^ This passive approach may lead to unfavorable consequences for the patients because pseudoprogression is not commonly expected.^7^ Unfortunately, no predictive factor of primary resistance disease (PRD) currently exists, and clinicians are forced to make treatment decisions without any quantitative information. For patients with PRD, the timely administration of other IO combination regimens might be better to avoid impacting a limited lifetime and unnecessary immune‐related adverse events (irAEs). This study aimed to evaluate the risk factors for primary resistance to NIVO+IPI in patients with mRCC. If patients likely to experience PRD by NIVO+IPI treatment could be identified, it could save precious time for the individual patients.

This study also aimed to retrospectively determine the clinical aspects of PRD in patients with mRCC treated with NIVO+IPI using a cohort of 120 patients from multiple institutes throughout Japan.

## 2 MATERIALS AND METHODS

### 2.1 Data collection and study cohort

This retrospective multi‐institutional study was approved by the review board of all participating institutes (ID: 2245 in the Institutional Review Board of Akita University Graduate School of Medicine). Informed consent was not required for this retrospective study. The inclusion criteria were patients who were treated with NIVO+IPI as a first‐line therapy for mRCC. Those with uncontrolled comorbidities were excluded. In total, 120 patients treated with NIVO+IPI for mRCC between August 2015 and January 2023 at 11 institutes were included. The clinical demographics and baseline characteristics of the enrolled patients were analyzed. mRCC was histologically verified in all included patients.

### 2.2 Definition of primary progressive disease

PRD was defined as a progressive disease diagnosed at the first imaging evaluation or clinical progression within 3 months of treatment initiation.

### 2.3 Study endpoints

The primary objectives were to compare the OS and progression‐free survival (PFS) between patients who showed PRD and those who did not show PRD. The objective response rate was described based on the development of PRD as a secondary objective. The risk factors associated with PRD were screened and confirmed using Cox regression analysis.

### 2.4 NIVO+IPI treatment

The criteria, dose, and schedule of NIVO+IPI consisted of four cycles of NIVO+IPI and subsequent nivolumab monotherapy until patients developed intolerant irAEs or disease progression was clinically confirmed. The treatment schedule did not strictly follow the drug guidance, and NIVO+IPI was administered at the discretion of each physician. All analyzed patients were categorized as being at intermediate or poor risk according to the IMDC.

### 2.5 Patient monitoring

The monitoring protocol was not unified and depended on the decision of each physician. We collected data, including demographic information, RCC histology, the number of metastatic sites, body mass index, Eastern Cooperative Oncology Group performance status, complete blood count with differential and platelet, biochemical profile, urinalyses, chest radiography platelets, and irAEs, from the patients' medical records. Neutrophil‐to‐lymphocyte ratio, platelet‐to‐lymphocyte ratio, and *systemic* immune‐*inflammation index* (calculated by [neutrophil × platelet]/lymphocyte) were investigated as candidate predictive markers for PRD. Lymph node swelling 1 cm or more detected by computed tomography (CT) scan in both regional and extra‐regional was diagnosed as lymph node metastasis (LNM). These parameters were re‐evaluated during therapy based on the decision of the attending physicians. Toxicity data were collected by investigators and coded according to the National Cancer Institute Common Toxicity Criteria version 2.0. Tumor responses were measured using CT scans and assessed using the Response Evaluation Criteria in Solid Tumors guidelines version 1.1. After starting the IO combination therapy, the admission and assessment schedule was considered carefully by the attending physicians on an individual basis. This report conforms to the STROBE guidelines.

### 2.6 Statistical analysis

OS was defined as the time interval from the first NIVO+IPI treatment to the date of death from any cause, with censoring at the last follow‐up. PFS was defined as the time from the first induction of immunotherapy to disease progression or date of death (whichever occurred first), with censoring at the time of the last follow‐up. The database record was closed upon patient death or at the final follow‐up. Data were expressed as the median and interquartile range (IQR), and statistical significance was set at *p* < 0.05. The frequencies of categorical variables were compared using the Pearson chi‐square or Fisher's exact test, whichever was appropriate, and the odds ratio (OR) was estimated in proportions. Survival curves were generated using the Kaplan–Meier method and compared using Cox proportional hazard regression analysis. The Cox hazard analysis was also applied to investigate hazard ratio (HR) and 95% confidence interval (CI) in univariate and multivariable analyses. Clinical factors were included in the multivariable analysis if their univariate *p*‐value was <0.1. All data were analyzed using the SPSS version 26.0 statistical software (SPSS Japan Inc.).

## 3 RESULTS

### 3.1 Patient characteristics

In total, 120 patients with mRCC were analyzed in this study, and their baseline demographics are shown in Table 1. The median age at NIVO+IPI initiation was 68 years (IQR, 61–73), and most patients were male (*n* = 86, 71.7%) and had clear cell histology (*n* = 104, 86.7%). Nephrectomy was performed for a limited number of patients (*n* = 47, 38.2%). Induction therapy was not completed in 48 patients (40%). Fifty‐seven patients (47.5%) were classified as intermediate risk based on the IMDC risk classification system, whereas 63 were classified as poor risk (52.5%). The number of metastatic organs was one in 30 patients (25%), two in 39 patients (32.5%), and three or more in 51 patients (42.5%). The distribution of treatment target sites at baseline was the lungs (*n* = 85, 70.8%), primary tumor site (*n* = 48, 40%), lymph nodes (LN; *n* = 44, 36.7%), bones (*n* = 36, 30%), liver (*n* = 29, 24.2%), brain (*n* = 15, 12.5%), adrenal glands (*n* = 12, 10%), pancreas (*n* = 10, 8.3%), pleura (*n* = 8, 6.7%), soft tissue (*n* = 8, 6.7%), and others (*n* = 7, 5.8%).

**TABLE 1:** Patient demographics.

Number of patients 120 (100%)	
Gender (%)	
Male	86 (72)
Female	34 (28)
Age (years)	
Median	68
IQR	61–73
Histology (%)	
Clear cell	104 (87)
Others	16 (13)
Prior nephrectomy (%)	
Yes	47 (39)
ECOG PS	
0 or 1	86 (71)
2 or more	27 (23)
Unknown	7 (6)
IMDC risk classification	
Intermediate	57 (47)
Poor	63 (53)
Observational period (months)	
Median	16
IQR	5–27
Treatment duration (months)	
Median	4.2
Range	2–9
Clinical stage at diagnosis of RCC (%)	
1	6 (5)
2	3 (2)
3	6 (5)
4	105 (88)
Number of target organs for treatment	
1	30 (25)
2	39 (32)
≥3	51 (43)
Site of metastasis and recurrence	
Lung	85 (71)
Primary site	48 (40)
Lymph node	44 (37)
Bone	36 (30)
Liver	29 (24)
Brain	15 (12)
Adrenal	12 (10)
Pancreas	10 (8)
Pleura	8 (7)
Soft tissue	8 (7)
Others	7 (6)
Best response	
CR	7 (5)
PR	42 (35)
SD	39 (33)
PD	26 (22)
Unknown	6 (5)
irAE	
Grade 1 or more	80 (67)
Grade 3 or more	47 (39)
Treatment completion	72 (60)
Reason for nivolumab discontinuation	
PD	60 (50)
AE	9 (8)
Still continue	42 (35)
Other reason	9 (8)
Steroid (PLS ≧ 40 mg) for irAEs	39 (33)

Abbreviations: AE, adverse event; CR, complete response; IMDC, International Metastatic Renal Cell Carcinoma Database Consortium; irAE, immune‐related adverse event; PD, progressive disease; PLS, prednisolone; PR, partial response; RCC, renal cell carcinoma; SD, stable disease.

### 3.2 Evaluation of response to treatment (including PRD)

The median follow‐up period for the whole cohort was 16 months (IQR, 5–27). All patients were followed up using the analysis. Nine patients were excluded from the analysis owing to the lack of evaluation of the best response to treatment. Twenty‐six of 111 patients (23.4%) were evaluated as having PRD based on progression within 3 months of treatment initiation (Table 2) according to imaging modality or clinical course. There were no treatment‐related deaths.

**TABLE 2:** Characteristics in patients showed response with primary resistance or disease controlled.

	All patients	Primary PD	Disease control	*p*‐Value
	(*N* = 111)	(*N* = 26)	(*N* = 85)	
Age (years)				
Median (IQR)	68 (61–73)	69 (58–73)	68 (62–75)	0.618
BMI (kg/m^2^)				
Median (IQR)	22.4 (20.6–25.2)	23.0 (20.8–25.6)	22.3 (20.5–24.9)	0.546
Sex (%)				
Male	82 (74)	23 (88)	59 (69)	0.073
Female	29 (26)	3 (12)	26 (31)	
Nephrectomy (%)				
Yes	44 (39)	9 (35)	35 (41)	0.649
No	67 (60)	17 (65)	50 (59)	
ECGO PS				
0 or 1	66 (59)	14 (54)	52 (61)	0.263
2 or more	40 (36)	12 (46)	28 (33)	
unknown	5 (5)	0 (0)	5 (6)	
Histology (%)				
Clear cell	97 (87)	20(77)	77 (91)	0.090
Non‐clear cell	14 (13)	6 (23)	8 (9)	
Clinical stage (%)				
1	6 (5)	0 (0)	6 (7)	0.222
2	3 (2)	0 (0)	3 (4)	
3	4 (5)	2 (8)	2 (2)	
4	98 (89)	24 (92)	74 (87)	
IMDC risk classification (%)				
Intermediate	52 (45)	9 (35)	43 (51)	0.182
Poor	59 (55)	17 (65)	42 (49)	
Number of treatment target organs (%)				
1	27 (39)	4 (15)	23 (27)	0.149
2	38 (30)	7 (27)	31 (36)	
3≤	46 (31)	15 (58)	31 (36)	
Target organ (%)				
Lung	79 (71)	21 (81)	58 (68)	0.322
Primary site	43 (61)	9 (35)	34 (40)	0.654
Lymph node	39 (38)	16 (62)	23 (27)	0.002
Bone	34 (31)	12 (46)	22 (26)	0.057
Liver	27 (24)	8 (31)	19 (22)	0.436
Brain	14 (14)	4 (15)	10 (12)	0.736
Adrenal	12 (10)	1 (4)	11 (13)	0.288
Pancreas	10 (8)	1 (4)	9 (11)	0.448
Pleura	8 (7)	2 (8)	6 (7)	0.678
Soft tissue	8 (7)	2 (8)	6 (7)	1.000
CRP (mg/L)				
Median (IQR)	1.1 (0.2–6.3)	5.0 (0.9–15.9)	0.9 (0.2–4.5)	0.008
NLR				
Median (IQR)	3.6 (2.4–5.9)	4.2 (3.6–9.5)	3.3 (2.3–5.2)	0.011
PLR				
Median (IQR)	225 (154–370)	261 (181–436)	221 (151–363)	0.255
SII				
Median (IQR)	931 (650–1698)	1305 (798–2672)	892 (644–1610)	0.052

Abbreviations: BMI, body mass index; CRP, C‐reactive protein; IMDC, International Metastatic Renal Cell Carcinoma Database Consortium; IQR, interquartile range; NLR, neutrophil lymphocyte ratio; PD, progressive disease; PLR, platelet lymphocyte ratio; SII, systemic immune inflammation index.

### 3.3 Adverse events

irAEs were observed in 76 patients (63.3%), detailed in Table 3. Of the 76 patients, 56 (50%) had Grade ≥3 severe irAE. Only five patients in the PRD group suffered from severe irAE because their treatment periods were shorter than those of other patients, which resulted in a low rate of irAE.

**TABLE 3:** The all reported immune‐related adverse events in our study.

irAE (%)	All grade	≧Grade 3
All event	76 (63)	56 (50)
Dermatitis	24 (20)	7 (6)
Hypophysitis	18 (15)	10 (8)
Hypothyroidism	14 (12)	2 (2)
Interstitial pneumonia	11 (9)	6 (5)
Liver dysfunction	11 (9)	6 (5)
Adrenal insufficiency	5 (5)	3 (3)
Destructive thyroiditis	4 (4)	3 (3)
Type I diabetes mellitus	3 (3)	3 (3)
Diarrhea	3 (3)	2 (2)
Trouble of intestine	3 (3)	2 (2)
Endocarditis	2 (2)	2 (2)
Electrolyte imbalance	2 (2)	2 (2)
Hypertension	2 (2)	1 (1)
General fatigue	2 (2)	1 (1)
Anemia	2 (2)	1 (1)
Uveitis	2 (2)	1 (1)
Arthritis	2 (2)	0 (0)
Fever	2 (2)	0 (0)
ALS	1 (1)	1 (1)
Pancreatitis	1 (1)	1 (1)
Kidney dysfunction	1 (1)	1 (1)
Others	5 (5)	1 (1)

Abbreviations: ALS, amyotrophic lateral sclerosis; irAE, immune related adverse event.

### 3.4 Treatment outcomes and survival rate

**FIGURE 1:**
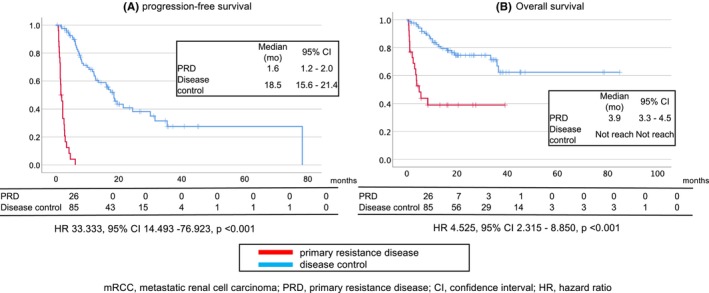
Kaplan–Meier curve of progression‐free survival (A) and overall survival (B) in patients with metastatic renal cell carcinoma based on whether they demonstrated primary resistance to nivolumab and ipilimumab treatment.

Patients who experienced PRD showed worse PFS (HR: 33.333, 95% CI: 14.493–76.923, *p* < 0.001) and OS (HR: 4.525, 95% CI: 2.315–8.850, *p* < 0.001) (Figure 1).

### 3.5 Analyses of factors associated with OS

The univariate analysis showed that high C‐reactive protein, high neutrophil‐to‐lymphocyte ratio, and LNM were associated with PRD (Table 1). Multivariable analysis showed that LNM (OR: 4.274, 95% CI: 1.075–16.949, *p* = 0.039) was an independent risk factor for PRD (Table 4).

**TABLE 4:** Predictive clinical valuable for primary progressive disease in mRCC patients treated with nivolumab plus ipilimumab analyzed by using univariate and multivariate logistic regression models.

Risk factor	Risk category	Univariate				Multivariable			
		OR	95% CI		*p*‐Value	OR	95% CI		*p*‐Value
			Lower	Upper			Lower	Upper	
CRP	0.5 or more	2.941	1.012	8.547	0.048	1.087	0.269	4.405	0.907
NLR	3.0 or more	4.032	0.876	18.519	0.074	2.282	0.097	53.770	0.609
SII	315 or more	5.155	1.067	25.000	0.041	5.952	0.284	125.000	0.250
Sex	Male	3.378	0.931	12.195	0.064	2.632	0.503	13.699	0.252
Histology	Non‐clear cell	2.890	0.898	9.259	0.075	2.801	0.509	15.385	0.236
Number of target organs for treatment	Three or more	2.375	0.971	5.811	0.058	1.091	0.305	3.902	0.893
Metastatic organ	Lymph node	5.405	2.110	13.889	<0.001	4.274	1.075	16.949	0.039
	Bone	2.457	0.987	6.098	0.053	2.874	0.891	9.259	0.077

Abbreviations: CI, confidence interval; CRP, C‐reactive protein; mRCC, metastatic renal cell carcinoma; NLR, neutrophil‐lymphocyte ratio; OR, odds ratio; SII, systemic immune inflammation index.

## 4 DISCUSSION

In this retrospective, multi‐institutional study, 23.4% of patients with mRCC demonstrated primary resistance to NIVO+IPI therapy. OS and PFS were worse in patients diagnosed with PRD. Positive LNM was a risk factor for PRD. To the best of our knowledge, this is the first report to analyze the clinical aspects of PRD in patients with mRCC treated with NIVO+IPI.

Clinical outcomes in patients with mRCC have improved consistently because of the introduction of effective treatment options such as IO combinations. In these IO combinations, NIVO+IPI is shown to be efficacious.^8^ Approximately 10% of patients accomplished complete disease remission, whereas more than 20% achieved durable responses and presented with a measurable disease without any progression for years.^5^ These patients were not expected to die of disease progression and thus were thought to be “cured.” This result convinced us to use NIVO+IPI with the objective to cure patients in instances of younger age, high tumor burden, and poor response to IO–TKI combinations. However, NIVO+IPI failed to achieve any clinical benefit at any significant rate.^4^ In a pivotal study, CheckMate 214, 20% of patients did not benefit from NIVO+IPI therapy, similar to the proportion of patients from the present study in whom NIVO+IPI was not efficacious. These patients showed poor survival, as was predicted. Furthermore, some difficulties exist in judging a PD after NIVO+IPI initiation in patients. The first is pseudoprogression, also called tumor flare.^6^ When an immune‐checkpoint inhibitor is used, there is usually a waiting period of at least 2 months to evaluate the treatment efficacy of the IO drug to distinguish it from pseudoprogression.^9^ Even this brief delay may be crucial for patients with PRD. The second is hyper or critical progression after NIVO+IPI administration.^10^ This unexpected reaction did not allow patients a chance to recover from the disease. These two aspects of NIVO+IPI treatment allowed for two distinct treatment strategies: positive selection of patients and avoidance. In the present study, we focused on patient avoidance, thus avoiding a patient in whom NIVO+IPI treatment is less likely to be beneficial. The irresponsible administration of NIVO+IPI without enough provisions tends to be detrimental for patients.

Some mechanisms underlying resistance to IO drugs have been investigated.^11^ However, the actual cause of these mechanisms remains unclear.^2^ Efficient IO treatment needs precise function and enough infiltrations by effector T cells in a tumor environment. Tumor resistance to IO therapy is observed when malignant cells impair T cells at different stages of their maturation or effector function.^12^ Understanding the biological pathway underlying malignant cells' escape from immune surveillance by T‐cell suppression will facilitate the selection of better candidates for IO drugs for RCCs.^13^, ^14^ Primary resistance to NIVO+IPI manifests in approximately 20% of patients with mRCC, leading to a poor prognosis.^5^, ^15^ Overcoming this unexpected response is essential to clarify the primary resistance mechanisms.

NIVO+IPI demonstrated less effectiveness against LNM in the present study. IO drug has been known for organ‐specific effects.^16^ Anti‐PD‐1 antibody plus lenvatinib therapy showed less effectiveness against unresectable hepatocellular carcinomas and LNM than against macrovascular tumor thrombi or lung metastases.^17^, ^18^ A similar result might be expected in thyroid cancer with LN involvement.^19^ Furthermore, advanced gastric cancer with LNM showed a favorable survival rate under nivolumab treatment.^20^ These distinct reactions to IO drugs could be due to biological differences among carcinomas and/or the immune status of infiltrating T cells in LNs. The lymphocyte subset in LN may be associated with a treatment result of the IO drug in patients with LN disease.^21^ Patients with LN diseases using the IO drug might experience a favorable outcome when effector T cells with PD‐1(+) CD8(+) dominantly infiltrate the tumors in the LN.^21^ In contrast, CD4(+) CD39(+) T cells are already divergent from exhausted T cells.^22^ These exhausted T cells are rarely reprogrammed, even given enough inhibition to the PD‐1 pathway.^23^ Infiltration of Treg cells may also be associated with the negative reaction of LNM.^24^ Another explanation of the distinct treatment consequence among cancers with LNM is the existence of tertiary LN.^25^ Infection‐induced cancer, such as gastric or cervical cancer, usually involves tertiary LNs during the chronic inflammatory phase of pre‐existing tumorigenesis. Established alternative LN can work similarly to native LN in recruiting effector T cells to the tumor microenvironment.^26^ This explains the favorable result in patients with gastric cancer with LN involvement.

This study had several limitations, including patient and treatment selection biases inherent in the retrospective design. The limited sample size is an additional limitation. However, we performed a Cox multivariable regression analysis to mitigate possible biases associated with confounders, which is the strength of the present study because the existence of LNM did predict PRD independently.

## 5 CONCLUSIONS

This retrospective study confirmed that PRD was associated with poor survival and showed that LNM was independently associated with PRD in patients with mRCC treated with NIVO+IPI as first‐line therapy. mRCC patients with LNM may benefit from IO combination treatments other than NIVO+IPI.

## AUTHOR CONTRIBUTIONS

**Kazuyuki Numakura:** Conceptualization (equal); data curation (equal); formal analysis (equal); funding acquisition (equal); investigation (equal); methodology (equal); project administration (equal); resources (equal); software (equal); validation (equal); visualization (equal); writing – original draft (equal); writing – review and editing (equal). **Yuya Sekine:** Data curation (equal); formal analysis (equal); writing – original draft (equal). **Shingo Hatakeyama:** Conceptualization (equal); data curation (equal); formal analysis (equal); writing – original draft (equal). **Yumina Muto:** Data curation (equal). **Ryuta Sobu:** Data curation (equal); writing – original draft (equal). **Mizuki Kobayashi:** Data curation (equal). **Hajime Sasagawa:** Data curation (equal). **Soki Kashima:** Data curation (equal). **Ryohei Yamamto:** Data curation (equal). **Taketoshi Nara:** Data curation (equal). **Hideo Akashi:** Data curation (equal). **Ryuji Tabata:** Data curation (equal). **Satoshi Sato:** Data curation (equal). **Mitsuru Saito:** Data curation (equal). **Shintaro Narita:** Data curation (equal). **Chikara Ohyama:** Supervision (equal); writing – review and editing (equal). **Tomonori Habuchi:** Conceptualization (equal); supervision (equal); writing – review and editing (equal).

## FUNDING INFORMATION

This study was partially supported by the Grants‐in‐Aid for Scientific Research, Japan (Grant No. 20K09553).

## CONFLICT OF INTEREST STATEMENT

The authors have no conflicts of interest.

## Data Availability

Data are available for bonafide researchers upon request from the authors.

## References

[1] Motzer RJ , Escudier B , McDermott DF , et al. Nivolumab versus everolimus in advanced renal‐cell carcinoma. N Engl J Med. 2015;373:1803‐1813. 26406148 10.1056/NEJMoa1510665 PMC5719487

[2] Zhuang TZ , Case K , Olsen TA , et al. Metastatic clear‐cell renal cell carcinoma in the era of immune checkpoint inhibitors: therapies and ongoing trials. Cancers (Basel). 2022;14:2867. 35740533 10.3390/cancers14122867 PMC9220801

[3] Ljungberg B , Albiges L , Abu‐Ghanem Y , et al. European Association of Urology guidelines on renal cell carcinoma: the 2022 update. Eur Urol. 2022;82:399‐410. 35346519 10.1016/j.eururo.2022.03.006

[4] Motzer RJ , Escudier B , McDermott DF , et al. Survival outcomes and independent response assessment with nivolumab plus ipilimumab versus sunitinib in patients with advanced renal cell carcinoma: 42‐month follow‐up of a randomized phase 3 clinical trial. J Immunother Cancer. 2020;8:e000891. 32661118 10.1136/jitc-2020-000891 PMC7359377

[5] Motzer RJ , Tannir NM , McDermott DF , et al. Nivolumab plus ipilimumab versus sunitinib in advanced renal‐cell carcinoma. N Engl J Med. 2018;378:1277‐1290. 29562145 10.1056/NEJMoa1712126 PMC5972549

[6] Taleb BA . Tumour flare reaction in cancer treatments: a comprehensive literature review. Anticancer Drugs. 2019;30:953‐958. 31348010 10.1097/CAD.0000000000000814 PMC6749970

[7] Hodi FS , Hwu WJ , Kefford R , et al. Evaluation of immune‐related response criteria and RECIST v1.1 in patients with advanced melanoma treated with pembrolizumab. J Clin Oncol. 2016;34:1510‐1517. 26951310 10.1200/JCO.2015.64.0391 PMC5070547

[8] Rini BI , Signoretti S , Choueiri TK , et al. Long‐term outcomes with nivolumab plus ipilimumab versus sunitinib in first‐line treatment of patients with advanced sarcomatoid renal cell carcinoma. J Immunother Cancer. 2022;10:e005445. 36549781 10.1136/jitc-2022-005445 PMC9791431

[9] Numakura K , Kobayashi M , Hatakeyama S , et al. Efficacy and safety of nivolumab for renal cell carcinoma in patients over 75 years old from multiple Japanese institutes. Int J Clin Oncol. 2020;25:1543‐1550. 32394047 10.1007/s10147-020-01693-y

[10] Lecis D , Sangaletti S , Colombo MP , Chiodoni C . Immune checkpoint ligand reverse signaling: looking back to go forward in cancer therapy. Cancers (Basel). 2019;11:624. 31060225 10.3390/cancers11050624 PMC6563035

[11] Lu X , Vano Y , Helleux A , et al. An enhancer demethylator phenotype converged to immune dysfunction and resistance to immune checkpoint inhibitors in clear‐cell renal cell carcinomas. Clin Cancer Res. 2022;29:1279‐1291. doi:10.1158/1078-0432.CCR-22-2133 36374555

[12] Chen DS , Mellman I . Oncology meets immunology: the cancer‐immunity cycle. Immunity. 2013;39:1‐10. 23890059 10.1016/j.immuni.2013.07.012

[13] Gide TN , Wilmott JS , Scolyer RA , Long GV . Primary and acquired resistance to immune checkpoint inhibitors in metastatic melanoma. Clin Cancer Res. 2018;24:1260‐1270. 29127120 10.1158/1078-0432.CCR-17-2267

[14] Davis RS . Roles for the FCRL6 immunoreceptor in tumor immunology. Front Immunol. 2020;11:575175. 33162991 10.3389/fimmu.2020.575175 PMC7591390

[15] Kido K , Hatakeyama S , Numakura K , et al. Comparison of nivolumab plus ipilimumab with tyrosine kinase inhibitors as first‐line therapies for metastatic renal‐cell carcinoma: a multicenter retrospective study. Int J Clin Oncol. 2021;26:154‐162. 33067647 10.1007/s10147-020-01797-5

[16] Numakura K , Horikawa Y , Kamada S , et al. Efficacy of anti‐PD‐1 antibody nivolumab in Japanese patients with metastatic renal cell carcinoma: a retrospective multicenter analysis. Mol Clin Oncol. 2019;11:320‐324. 31341623 10.3892/mco.2019.1887 PMC6636209

[17] Lu LC , Hsu C , Shao YY , et al. Differential organ‐specific tumor response to immune checkpoint inhibitors in hepatocellular carcinoma. Liver Cancer. 2019;8:480‐490. 31799205 10.1159/000501275 PMC6883443

[18] Huang C , Zhu XD , Shen YH , et al. Organ specific responses to first‐line lenvatinib plus anti‐PD‐1 antibodies in patients with unresectable hepatocellular carcinoma: a retrospective analysis. Biomark Res. 2021;9:19. 33743822 10.1186/s40364-021-00274-z PMC7981986

[19] Gong Z , Jia H , Xue L , et al. The emerging role of transcription factor FOXP3 in thyroid cancer. Rev Endocr Metab Disord. 2022;23:421‐429. 34463908 10.1007/s11154-021-09684-8

[20] Formica V , Morelli C , Patrikidou A , Shiu KK , Roselli M , Arkenau HT . Lymph node‐only metastatic gastric/gastroesophageal junction cancer and efficacy of immunotherapy. Gastric Cancer. 2020;23:1107‐1108. 32424650 10.1007/s10120-020-01084-2

[21] Rawson RV , Adhikari C , Bierman C , et al. Pathological response and tumour bed histopathological features correlate with survival following neoadjuvant immunotherapy in stage III melanoma. Ann Oncol. 2021;32:766‐777. 33744385 10.1016/j.annonc.2021.03.006

[22] Balanca CC , Salvioni A , Scarlata CM , et al. PD‐1 blockade restores helper activity of tumor‐infiltrating, exhausted PD‐1hiCD39+ CD4 T cells. JCI Insight. 2021;6:e142513. 33332284 10.1172/jci.insight.142513 PMC7934837

[23] Pauken KE , Sammons MA , Odorizzi PM , et al. Epigenetic stability of exhausted T cells limits durability of reinvigoration by PD‐1 blockade. Science. 2016;354:1160‐1165. 27789795 10.1126/science.aaf2807 PMC5484795

[24] Nunez NG , Tosello Boari J , Ramos RN , et al. Tumor invasion in draining lymph nodes is associated with treg accumulation in breast cancer patients. Nat Commun. 2020;11:3272. 32601304 10.1038/s41467-020-17046-2 PMC7324591

[25] Helmink BA , Reddy SM , Gao J , et al. B cells and tertiary lymphoid structures promote immunotherapy response. Nature. 2020;577:549‐555. 31942075 10.1038/s41586-019-1922-8 PMC8762581

[26] Yamakoshi Y , Tanaka H , Sakimura C , et al. Immunological potential of tertiary lymphoid structures surrounding the primary tumor in gastric cancer. Int J Oncol. 2020;57:171‐182. 32319601 10.3892/ijo.2020.5042 PMC7252463

